# Adaptation and Acceptability of a Low-Intensity Cognitive Behavioral Therapy App to Support Low Mood and Worry Management in Female Forces Veterans: Mixed Methods Study

**DOI:** 10.2196/84365

**Published:** 2026-04-29

**Authors:** Paul Farrand, Andy Bacon, Melika Janbakhsh, Natalie Flay, Elizabeth Turnbull, Jonathan Baker

**Affiliations:** 1 Clinical Education, Development and Research (CEDAR), Psychology, Faculty of Health and Life Sciences, University of Exeter, Perry Road, Exeter, EX4 4QG, United Kingdom, 44 7533192764; 2 Westminster Centre for Research in Veterans, University of Chester, Cheshire, United Kingdom; 3 Iona Mind Inc, Wilmington, DE, United States

**Keywords:** female, veterans, armed forces, adaptation, acceptability, low-intensity CBT, Ecological Validity Framework, mixed methods

## Abstract

**Background:**

Mental health help-seeking barriers experienced by female forces veterans result in them being underserved and underrepresented. Efforts are therefore required to adapt interventions for female veterans to enhance acceptability and maximize engagement. Given a smaller number and wider geographical distribution of female veterans, targeting adaptation efforts at a digital mobile phone app based on cognitive behavioral therapy (CBT) has potential for greatest impact to improve access to a scalable evidence-based psychological therapy.

**Objective:**

This study aimed to examine the adaptation of a low-intensity CBT app to support low mood and worry management in female forces veterans and examine acceptability and usability.

**Methods:**

Using a mixed methods methodology, this study comprises a focus group of female forces veterans to inform adaptation with extracted themes used as the basis of an adaptation framework. Following adaptation, a wider sample of female veterans was recruited to use the app and complete the mHealth App Usability Questionnaire to determine acceptability, usability, and usefulness.

**Results:**

Two main areas were identified as requiring adaptation to maximize acceptability and usability. While using imagery and quotes to reflect the armed forces was initially found helpful to initiate engagement, it was considered that continued reference to the armed forces should be dropped when progressing through the app. Most app features were found acceptable; however, adaptations were requested to the content and structure of signposting information, navigation, and the way progress was monitored. No adaptations were required, however, regarding the CBT techniques used, with specific app features motivating engagement. Following the adaptation, there were good levels of acceptability, usability, and usefulness.

**Conclusions:**

Involving female forces veterans as part of an intervention adaptation process has promise to improve acceptability and engagement with a digital CBT mobile phone intervention. Ensuring that the intervention represented the transition from serving to female forces veteran is of particular significance in enhancing acceptability.

## Introduction

Increased efforts are being made to recruit females into the UK Armed Forces who currently account for 11.5% of Regular and 16% of Reservists [1]. However, while government policy is seeking to increase this number to 30% by 2030, intake dropped by 1% in 2024 [1]. Despite significant efforts being made to integrate females into active service, female forces veterans remain underserved [2]. Difficulties are experienced because of support systems primarily developed for males with limited tailoring to the distinct needs of female forces veterans [3].

Both male and female forces veterans face barriers accessing mental health support such as stigma, concerns they will not be understood by health care providers, and a culture of stoicism [4]. However, female veterans frequently report additional gendered adversity, such as military sexual trauma and emotional bullying [5]. Furthermore, they may need to navigate tensions between military culture and their feminine identity [6]. In other words, managing disparities between military culture and feminine norms, discrimination experienced both during and after service, lack of awareness, and particularly branding that reinforces male veteran stereotypes [7] are additional barriers to accessing mental health support faced by female forces veterans.

These barriers can, in turn, shape female forces veterans’ help-seeking behavior [8]. Reports indicate that while female forces veterans are more likely to seek statutory adult mental health services than male veterans, they are less likely to access veteran-specific services [9]. Consequently, female veterans’ lower engagement with veteran-specific services can limit their access to tailored support addressing areas such as finance, housing, employment, and welfare [10].

Additionally, compared with male veterans (16.7% and 13.5%), the prevalence of depression and anxiety among female veterans is 25.8% and 25.5%, respectively [11]. Experiencing these stressors during transition into civilian life is of particular concern as this is recognized as a particularly vulnerable period [12]. During this transition, female forces veterans are required to negotiate identity changes, gender-related, and societal expectancies to fit with feminine norms and practical difficulties related to housing and employment [13]. Together, the reduced utilization of veteran-focused mental health services, accompanied by higher rates of depression and anxiety experienced by female forces veterans, may contribute to the existing treatment gap [14]. This is of concern, given a higher prevalence of common mental health difficulties amongst female forces veterans [15].

To reduce the treatment gap, low-intensity cognitive behavioral therapy (CBT) has been implemented in statutory adult mental health services, such as NHS (National Health Service) Talking Therapies for anxiety and depression [16]. Low-intensity CBT improves access via flexible and scalable goal-oriented and time-limited self-help interventions [17]. The approach has already been demonstrated to resonate with values associated with self-management held by the armed forces [18], with flexibility more likely to enhance help-seeking and engagement [19]. Furthermore, acceptability has been demonstrated where intervention adaptations are made to tailor language and imagery to target veterans [20].

Low-intensity CBT also provides greater ability to enhance flexibility through digital delivery [21]. This is particularly important for armed forces veterans, given the growing acceptance of digital mental health support [22]. However, while mobile apps are being developed, for example, to promote recognition of mental health problems and support help-seeking among armed forces veterans [23], they remain largely biased toward male veterans. Challenges to generalizability, therefore, endure, resulting in female forces veterans at risk of remaining underserved [24].

Using a mixed methods methodology [25], this study reports on the adaptation of the Iona app [21] for female forces veterans. Adopting a systematic approach, the Ecological Validity Framework [26] (EVF) serves to guide culturally sensitive adaptations for specific groups by considering 8 dimensions: language, persons, metaphors, content, concepts, goals, methods, and context. Making adaptations to these dimensions offers promise to maximize acceptability to improve engagement and access to a scalable mental health approach [27].

## Methods

### Intervention

**Figure 1.**
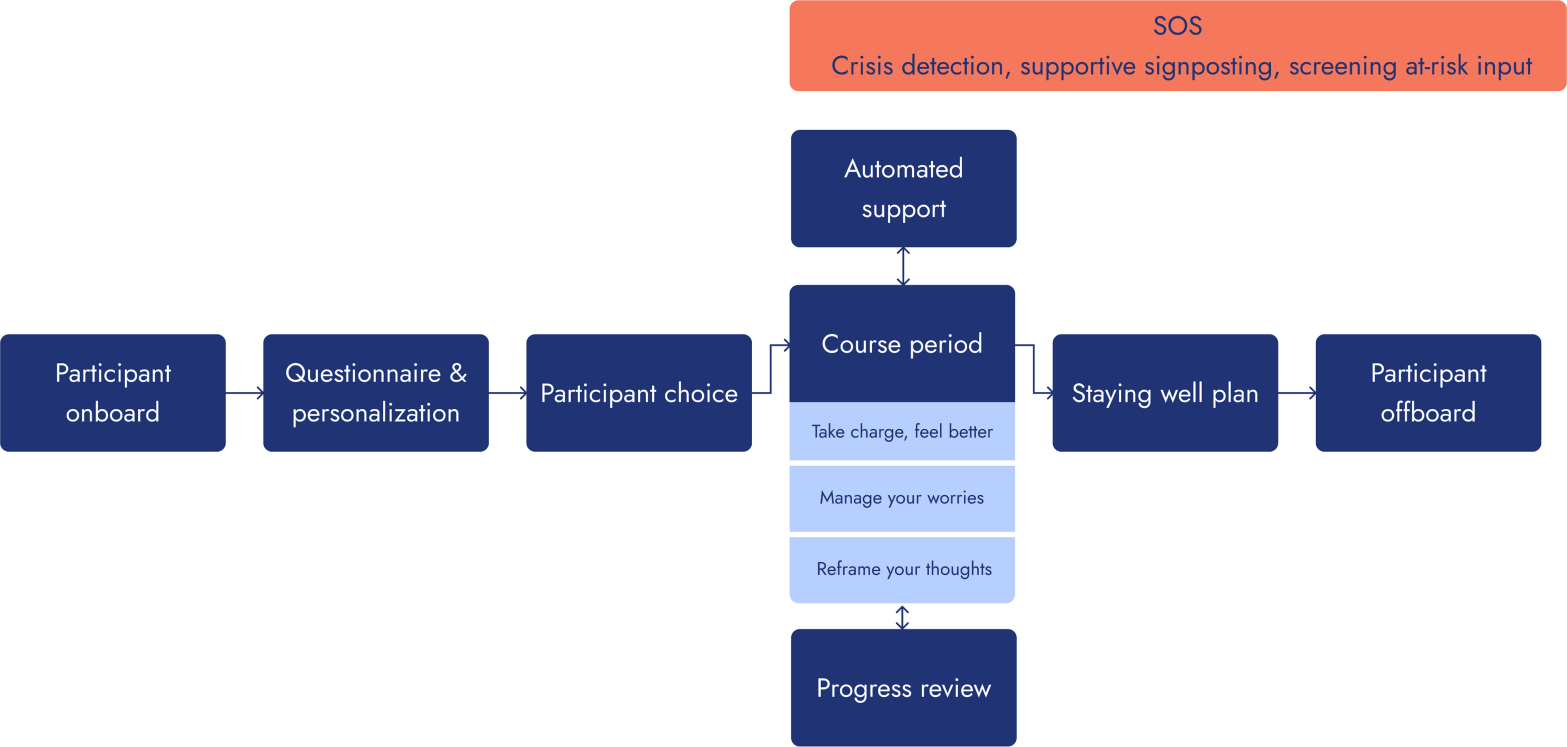
User flow through the Iona app.

The Iona Mind app (Iona) for the management of mild to moderate low mood [28] or excessive worry [21] informed the intervention adopted for adaptation (see Figure 1 for user flow through Iona).

After onboarding, the user is asked to complete the Patient Health Questionnaire-8 [29] and Generalized Anxiety Disorder-7 Scale [30] patient-reported outcome measures to assess the severity of low mood and worry symptoms, respectively. They then engage with an interactive CBT Five Areas model [31] before progressing through the intervention-specific factor techniques presented as 6 educational modules. Where the user scores mild to moderate symptom severity for both low mood and worry symptoms, they are directed to the technique corresponding with the highest symptom severity. Where symptom severity is identical, however, users are recommended the technique for low mood, although choice still can be exercised. An “SOS” button is available on every screen for users who either score within the severe range during onboarding or show signs of deterioration during use. If the person is seeking immediate help, the “SOS” page contains NHS Emergency contact details.

Where treatment for low mood is recommended, the user is introduced to the behavioral activation [32] or cognitive restructuring [33] techniques derived from low-intensity CBT [34]. Alternatively, where symptoms associated with worry are prominent, the Worry Management technique is proposed [21]. To facilitate choice, a description of each technique is available on request. Based on the evidence base, all techniques are informed by protocols recommended by the National Institute for Health and Care Excellence, Behavioral Activation [35,36], alongside Cognitive Restructuring for low mood [37] and Worry Management for excessive worry [38]. All techniques are included within the most commonly adopted written self-help interventions [39], used by NHS Talking Therapies for anxiety and depression services [40]. Engagement with the app is maintained through an omnipresent chatbot to establish a “therapeutic approach” [41]. This promotes collaborative empiricism to encourage the user to explore and reflect upon outcomes arising from engagement for themselves [42].

### Intervention Adaptation

The EVF [26] was adopted to systematically inform adaptation from the original Iona app to that specifically for Iona Ex-Servicewomen (IonaXSW). The app was later currently called “Iona Female Forces Veterans” (IonaFFV) based on results from phase 2. To ensure that cultural and contextual factors associated with the female forces veteran’s community are represented, the adaptation process focused on dimensions of the EVF [43] related to language, metaphor, context, and content. While dimensions related to concepts and methods were also adapted with respect to language, the theoretical rationale and technique-specific factors maintained high fidelity to the underlying CBT model. These dimensions were used to explicitly inform the focus group topic guide to ensure that structure was provided to ensure adequate coverage.

### Adaptation Process

**Figure 2.**
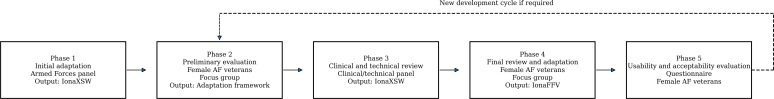
Adaptation process. AF: Armed Forces; IonaFFV: Iona Female Forces Veterans; IonaXSW: Iona Ex-Servicewomen.

The adaptation process comprised 5 phases to adapt Iona and examine its acceptability for female forces veterans (Figure 2). Across the 5 phases, 4 panels were established to provide different perspectives and contributed in varied ways with each panel building upon the work of the others [44].

During the adaptation process, an Armed Forces Panel was established to guide the initial adaptations, focusing on the context and content dimensions of the EVF. Phase 1 aimed to tailor the Iona app for female forces veterans and support the development of IonaXSW. After initial adaptations, a female forces veterans panel used their lived experience to refine the app further in phase 2. Data were analyzed to create an adaptation framework, which confirmed acceptable phase 1 adaptations and identified areas requiring additional modification. The framework was then provided to the clinical or technical panel in phase 3, who made further changes to develop the IonaFFV app. In phase 4, the app was returned to the same female forces veterans panel from phase 2 to assess the acceptability of the adaptations and ensure that changes based on the adaptation framework were effectively implemented. Finally, in phase 5, the IonaFFV app was released online to a wider group of female forces veterans to explore acceptability.

### Methodology

#### Study Design

To adapt the Iona app to maximize acceptability for female forces veterans, an exploratory sequential mixed methods research design comprising focus groups (phases 2 and 4) and a questionnaire to examine acceptability and usability (phase 5) were adopted [45].

##### Phase 1

###### Participants

The Armed Forces Panel comprised a male armed forces officer veteran who held senior NHS roles associated with armed forces veterans and study author (AB), a female officer forces veteran holding national roles in veterans’ charities, a male NHS Talking Therapies for anxiety and depression [16] armed forces veteran service lead, and a female regional commissioner for armed forces veterans.

###### Recruitment

Opportunistic sampling [46] based on personal contacts was held by AB. Participants contacted AB with preferences to organize a meeting time. The panel comprised 5 female forces veterans, 3 aged between 45 and 54 years and 2 aged between 35 and 44 years. Members of the panel had served in the Army (n=3), Royal Navy (n=1), and Royal Air Force (n=1).

###### Methods

Panel members were provided with a link to the unadapted Iona app and asked to review it before the meeting. A 1-hour online meeting, facilitated by AB and recorded for analysis, was held to gather initial feedback on areas requiring adaptation to better meet the needs of female forces veterans. Following the meeting, the recording was transcribed by a researcher (NF), who also made preliminary notes to develop an initial list of suggested adaptations. This list was then reviewed and refined in discussion with another researcher (PF). Once agreed upon, the final list of general adaptations was sent to the Iona app developer (JB) to create IonaXSW.

### Phase 2

#### Participants

The panel comprised 5 female forces veterans, 3 aged between 45 and 54 years and 2 aged between 35 and 44 years. Participants had served in the Army (n=3), Royal Navy (n=1), and Royal Air Force (n=1).

#### Recruitment

The female forces veterans panel, which served as the focus group, was recruited using snowball nonprobability convenience sampling [47]. Study advertisements, including a link to participant information, were initially posted on social media platforms that engage female forces veterans. Individuals who provided consent and contacted the researcher were invited to share the advertisement on their own social media accounts to further extend recruitment. Interested participants then indicated their availability for the focus group, which was scheduled with the first 5 participants for whom a suitable date could be confirmed. Participants were reimbursed only for travel expenses to attend the focus groups.

#### Methods

Prior to the focus group, all members were sent a link to IonaXSW and asked to work through it for 4 weeks, with support to download provided by the researcher if requested. Based on the focus group schedule informed by dimensions of the EVF, a 3-hour audio-recorded focus group was held face-to-face facilitated by 2 members of the research team (PF and NF). A reflective journal was used to log the progress, obstacles, and success encountered running the focus group [48]. The topic guide comprised open-ended questions to gain an initial impression of IonaXSW, address each dimension covered by the EVF [43], and explore the ability to use and engage with each app component. Following the focus group, the audio recording was transcribed and analyzed to inform a second adaptation framework.

#### Analysis

The focus group discussion was transcribed by NF with the transcript uploaded into NVivo (version 12.0; QSR International) to support manifest content analysis [49]. Analysis was based on articulated data [50] where NF read the transcript, coded meaning units using line-by-line inductive coding, and developed a codebook. During coding, concepts and important portions of raw text were recorded with a word or short phrase used to represent the meaning of a specific text segment. To develop a deeper and more nuanced understanding of the findings, the codebook was then discussed with PF with variations in coding discussed and refined until consensus was reached. This resulted in new codes being added until new concepts emerged, or similar concepts were grouped together to form categories and subcategories. To enhance trustworthiness, field notes and audit trails were taken to ensure that preconceived perspectives were not imposed on the data, with a checklist adopted to inform all phases of analysis [51].

### Phase 3

The clinical or technical panel comprised a male app developer (JB) for Iona Mind, and a male expert (PF) in low-intensity CBT apps who holds national expert advisory positions in low-intensity CBT and digital technologies. They used the adaptation framework developed in phase 2 to develop IonaFFV. The panel ensured that all adaptations maintained fidelity with the underlying low-intensity CBT clinical protocols, and software remained conformant to appropriate software requirements.

### Phase 4

#### Overview

Once adapted, the same participants who comprised the female forces veterans focus group in phase 2 were sent a link to IonaFFV and asked to engage with it for 4 weeks before participating in a second focus group. The topic guide for this session was designed to gather overall impressions of IonaFFV and to evaluate how effectively the adaptations proposed in phase 2 had been implemented, with reference to each dimension of the EVF. In addition, selected questions from previous topic guides on low-intensity CBT techniques [52] were incorporated to explore participant perceptions of different app components and their support preferences.

#### Analysis

Consistent with phase 2, the focus group discussion was transcribed and uploaded into NVivo (version 12.0) to support manifest content analysis [49]. Again, trustworthiness was established through discussions between NF and PF to reach consensus in coding [51].

### Phase 5

#### Participants

A total of 21 participants completed the demographic questionnaire, with a mean age of 49 years. Participants had left the armed forces between 1 and 43 years previously ( x̄=18.71), although 1 participant did not report their age (Table 1). The majority identified as female (n=20, 95%), White British (n=20, 95%), and as having served as a regular in the Navy (n=11, 52%).

**Table 1.** Participant demographics.

Characteristics	Frequency
Age range (years), n (%)	
25‐34	1 (5)
35‐44	5 (25)
45‐54	7 (35)
55‐64	5 (25)
65‐74	2 (10)
Gender, n (%)	
Female	20 (95)
Nonbinary	1 (5)
Ethnicity, n (%)	
White British	20 (95)
White and Black African	1 (5)
Service, n (%)	
Navy (regular)	11 (52)
Army (regular)	8 (38)
RAF^a^ (regular)	1 (5)
Navy (reserve)	1 (5)
Months served	
Average	144.4
Minimum	12
Maximum	372
Reason for discharge, n (%)	
Planned	14 (67)
Unplanned (medical, compulsory, and family related)	7 (33)
Deployed, n (%)	
Yes	14 (67)
No	7 (33)
Years since left service, n (%)	
Average	18.71
Minimum	1
Maximum	73

a RAF: Royal Air Force.

#### Recruitment

Permission was obtained to promote a study advertisement on the social media platforms used by charitable and community organizations providing support to British female forces veterans. Additionally, members of the female forces veterans focus group posted the study advertisement through relevant social media platforms they had membership and sent it to personal contacts. The study advertisement stated that they should be female forces veterans aged 18 years or older, not currently receiving mental health support, and had access to a smartphone with internet access. Potential participants consented online and were automatically sent a link to download IonaFFV with onboarding details. To promote recruitment, potential participants were offered the opportunity to enter a prize draw for a £100 (US $133) electronic voucher.

#### Methods

Following download, participants were asked to trial the app for 2 weeks, after which they were sent a link to complete the stand-alone patient version of the mHealth App Usability Questionnaire (MAUQ) on the Qualtrics platform [53]. This measure adopts 18 items to explore usability and acceptability across 3 subscales: Ease of Use, Interface and Satisfaction, and Usefulness. However, as IonaFFV was used in a research study focused on adapting an app for female forces veterans with participants who did not present with depression or anxiety and developed for use outside of health care services, it was considered that 3 questions were inappropriate. Consequently, “I would use this app again,” “The app improved my access to healthcare services,” and “The app helped me manage my health effectively” were removed.

Participants also completed a series of Iona-related questions. These included four 7-point Likert scale items, added after phase 4, to assess participants’ views on language, signposting information, and the SOS button. In addition, participants responded to a separate 7-point Likert scale question regarding human support. Those who selected Strongly Agree, Agree, Somewhat Agree, or Neither Agree nor Disagree were directed to answer 4 follow-up multiple-choice questions exploring different aspects of receiving human support alongside the app; multiple responses were permitted. At the end of the survey, all participants were invited to provide additional comments.

#### Analysis

For each subscale of the MAUQ, responses to the relevant items were averaged per participant and reported as median (IQR). All items were rated on a 7-point Likert scale, ranging from 1 (Strongly Agree) to 7 (Strongly Disagree). For analysis of the subscale items, responses of 1-3 were classified as agreement, 4 as neutral, and 5-7 as disagreement [54]. Percentages for each response category are reported for each question. 

Additional questions examining attitudes toward specific adaptations to IonaFFV arising from phase 4 were rated using 7-point Likert-scale questions alongside 4 multiple-choice questions regarding preferences regarding type of human support. The Likert-scale questions ranged from 1 (Strongly Agree) to 7 (Strongly Disagree), with means and SDs calculated for each question. Proportions (%) were reported for the multiple-choice questions, with a multiple response frequency analysis conducted to examine the distribution of selected options regarding preferences for different aspects of human support [55]. As participants could select up to 6 responses for 3 questions and up to 5 for 1 question, frequencies and percentages were calculated for each response option.  

### Ethical Considerations

Ethical approval was obtained for the focus groups (reference: 2611056) and online acceptability (reference: 5930558) studies from the University of Exeter psychology department. To ensure informed consent, all participants were provided with participant information sheets, which included information on the aims of the study, right to withdraw, data storage, and management processes. Participants were informed that their participation was entirely voluntary, and they could withdraw from the study without any negative consequences. No identifying information about participants is included. All data were reviewed to ensure that individuals cannot be identified, and no materials containing identifiable features were used. As all data were anonymized prior to analysis, participant consent for publication of identifying information was not required. Phases 2 and 4 focus group participants were offered compensation for travel costs, and phase 5 questionnaire participants were offered the opportunity to enter a prize draw for a US $133 electronic voucher.

## Results

### Overview

Overall, feedback provided by the female veterans focus group combined with responses added to the free-text response box included within the MAUQ [53] questionnaire administered in phase 5 highlighted that IonaFFV was well received. Awareness that the app had been adapted for female forces veterans was positively responded to.

*I’m still using it. It has helped a lot in the last 6 weeks or so. Please don’t take it away. I can dip in when I'm finding things tough and knowing it’s for female veterans is comforting*.

Adaptations appeared to promote continued engagement with IonaFFV as participants worked through each module.

*It made me want to continue to see what was coming next and where it was going and all that*.

However, there was 1 disconfirming case reported on the free response text box.

*The suggested activities are not relevant when you are actually struggling. I doubt anyone who has had an actual mental health episode was consulted. The activities are ok for when feeling a bit low, but not when you can't get out of bed and are struggling to function*.

### Phases 2 and 4

Analysis is presented in 2 parts. First, reflecting the attitudes expressed toward IonaXSW by the focus group during phase 2, alongside preferences regarding support. Second, attitudes expressed in phase 4 regarding the extent to which adaptations have been successfully implemented during phase 3 to create IonaFFV, alongside identifying outstanding areas requiring further adaptation. Categories identified as emerging were associated with “Context,” “App features,” and “No adaptations needed” (Table 2).

**Table 2.** Categories and subcategories associated with adaptation.

Armed forces context	App features	No adaptations required
Label	SOS page	CBT^a^ model
Graphics	Navigation	Contextual factors
Quotes	Monitoring progress	Mapping users
Avatar	N/A^b^	N/A

a CBT: cognitive behavioral therapy.

b N/A: not applicable.

### Armed Forces Context

This category captured the attitude of participants toward features of the Iona app that have been adapted to represent female forces veterans. These were grouped into label, graphics, quotes, and avatar.

#### Label

All participants in the focus group expressed disapproval toward using the “ex-servicewoman” label that was initially proposed by the Armed Forces Panel during intervention adaptation phase 1. They highlighted that this label does not accurately represent who they are and is not consistent with how they identify.

*I really don’t like the term ‘ex-servicewoman’. I don’t want to be viewed as ‘ex’ anything, but rather who I am now. I’m a female veteran and that’s what I would like to be called*.

While it was agreed that the term “ex-servicewoman” was not appropriate, there was wider discussion among participants regarding a preference for what other labels should be used. While all focus group members preferred the term “veteran” to “ex-servicewomen,” further discussion highlighted that some still considered it to be too vague.

*I don’t like veteran because it doesn't stipulate what you're a veteran of. You could be a veteran of working in an ice cream van*.

Participants further discussed using the term “female” versus “women” and armed forces, forces, or military. Consensus was finally reached regarding the term “female forces veteran.” Discussions around the language adopted here highlight the importance of this EVF dimension when undertaking adaptations for female forces veterans.

#### Graphics

**Figure 3.**
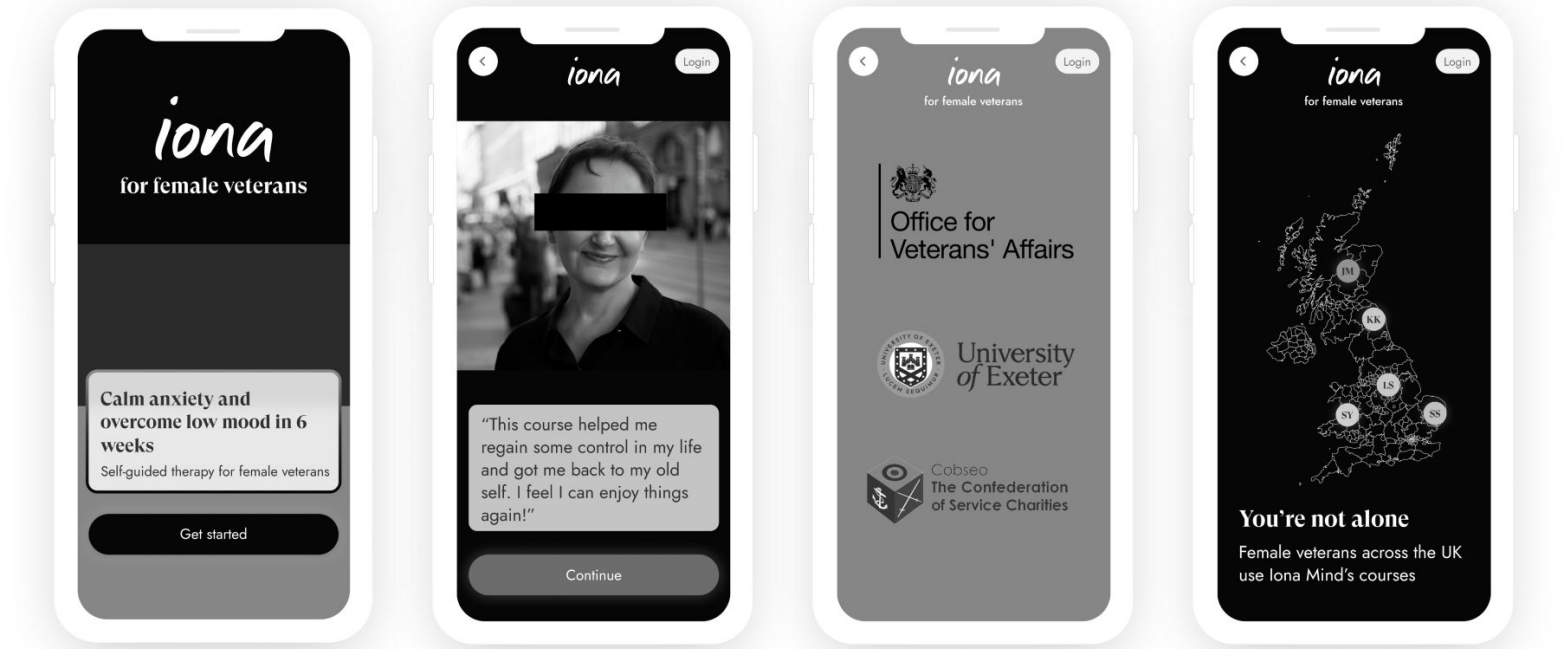
Adaptations to Iona Female Forces Veterans.

Participants expressed a complex set of attitudes toward the use of imagery related to the armed forces. There was general acceptance toward using the triservice colors representing the army, navy, and air force. Participants agreed that having the triservice color enhances the immediate association of the app with the armed forces (Figure 3A).

*Keep the armed forces colors because people with military connections will pick up on that*.

However, it was considered that using armed forces graphics was justified only when restricted to the start of the app. Participants emphasized that as they progressed through the app, the focus of the content needed to shift toward their identity as female forces’ veterans. Several participants also discussed that using these graphics within IonaXSW was extensive and could potentially lead to the app being considered “too official,” as this was a military platform. It was considered that this could lead to female forces veterans being reluctant to engage initially or maintain engagement with the IonaXSW app.

*If I could kind of have the benefit of, like, working through the app but without it feeling like too official, so using the image less so it’s like ‘this is unofficial’, you know, then maybe that*.

Another concern participants expressed regarding overusing armed forces–related graphics was that this could result in the app being seen as developed for male rather than female veterans. That is, participants recognized a lack of female veterans’ representation in the app.

*And I think I just worry that if it’s all branded, then it’s kind of, it’s like a bloke thing*.

#### Quotes

All participants agreed that using quotes from female forces veterans within IonaXSW was a good addition to promote motivation and continued engagement. They also agreed that locating these quotes near the start of the app was effective (Figure 3B). However, participants proposed that using quotes throughout IonaXSW should not be limited to the armed forces only but adapted to recognize the transition from the armed forces to civilians.

*The idea of using quotes was good, however they should not be military because you are transitioning out of the force and want to blend in*.

One solution suggested by a participant was to facilitate the personalization of quotes by enabling users to add their own.

*I think it’s quite nice* [to add your own quotes] *and you can go back and then have a look at, and, you know, read over them and think oh, actually yeah*.

Consistent with this solution, one participant suggested adding a tab specifically designed to enable users to add their own quotes and organize them into different categories.

*The motivational quotes are great. There should be a tab full of them with space to enter your own on a pinboard of gratitude, motivation, inspiration, and positivity*.

However, one participant noted that the user may need to exercise caution when allowing others to see their app with personalized quotes, given the unique armed forces sense of humor which could potentially cause offense to others.

*What you have to remember is the forces have got their own sick sense of humor and I think sometimes our morbid sense of humor comes, comes through, and it is again a very fine line*.

#### Avatars

Within phase 2, the female forces veterans were unanimous in their dislike of the avatars chosen to introduce the modules included within IonaXSW. Participants expressed concern that the avatars did not appropriately capture the context dimension of the EVF. Although the appearance and voice adopted for the avatar were initially chosen in phase 1 by members of the Armed Forces Panel to be representative of female forces veterans, this was not considered to be the case in phase 2.

*You look at them* [the avatar] *and you think, ‘Actually, you don’t look like you’ve ever served’. You know the person that you’re looking at has to be believable as well I think*.

However, when the same voice was used without the accompanying avatar to introduce different components of the CBT techniques, only the audio was appreciated.

*I liked the sound of the audio reading it* [technique components] *out, but would prefer not to see the face and just listen to it*.

### App Features

App features refer to attitudes held regarding general features included within IonaXSW to support help seeking, aid navigation and usability, and motivate continued engagement.

#### SOS Page

Participants highlighted the importance of having contact details for organizations to manage risk or access wider sources of armed forces–specific support. To aid navigation, attention was directed toward providing a relevant structure. This involved moving the helplines to manage high risk provided at the top, followed by Veterans Welfare and Female Veterans Support. Participants also discussed exclusion of any contact details that may become out-of-date.

*It’s very important to ensure that it* [link] *works and that those listed don't give users what’s termed as the “dockyard run around,” where they take your details but ultimately don't help you and pass you on*.

Consequently, links to regional organizations were excluded, and contact details for NHS Op COURAGE provided in the event details for regional support were requested. Some participants also recognized that not all users may be aware of the services offered by included organizations. It was therefore suggested to “include a brief description of what each charity provides.”

#### Navigation

Overall, participants reported IonaFFV as being easy to engage with and that progress through the educational modules was straightforward.

*Generally, I found it easy to move through the different parts* [of IonaFFV] *and get to where I wanted to go*.

However, uncertainty was expressed regarding the ability to navigate between the different low-intensity CBT techniques if they wanted to.

*Can, I ask, is there a back button? And you know if, if, at the moment, I'm going down one course* [low-intensity technique]*, because that’s what I selected, but can you go back to select another one? I haven't quite found that out*.

#### Monitoring

Features adopted in IonaFFV to support users in recognizing their progress and achievements after completing specific tasks were generally well received by participants in phase 4.

*I do like having the progress shown, and the praise for completing things*.

While participants appreciated knowing when a module had been completed, the use of graphics representing fireworks and confetti, along with the collection of badges to represent completion, was considered inappropriate for the target audience.

*Get rid of the fireworks ‘pop up’. It’s a bit patronizing; but yes, to having something that recognizes your achievement. Likewise, the concept of getting badges is great, but the look is a bit childish and patronizing*.

### No Adaptations Needed

Three main screens and interactive functionality were positively responded to by all focus group members in phase 2 and did not need further adaptation. This potentially indicates that initial adaptations in these areas made to accommodate female forces veterans were compatible with the content, methods, and context dimensions of the EVF.

#### CBT Model

Participants identified the “Here and Now” CBT model as helping the user understand the way in which a mental health difficulty can affect different areas of their life.

*I liked the diagram* [CBT model] *where all the different areas associated with a problem were linked together. It helped show how different things are linked and sorting one out by using a treatment* [CBT technique] *can help the others*.

Additionally, being able to complete the CBT model themselves was also found helpful. This enabled participants to personalize the app to their experience and motivated them to remain engaged.

*I liked that you filled in the boxes for yourself, so it reflected the problems you were having, and this made it more likely you wanted to carry on using it*.

#### Enhancing Contextual Factors

All participants agreed that presenting a screen with the names of trusted armed forces veterans’ organizations and a university at the start of IonaFFV engagement helped them establish a sense of trust and raised their expectations regarding the app’s effectiveness (Figure 3C).

*Seeing that screen with the armed forces and University logos was good as it made me feel I could trust the app and that it may help*.

#### Mapping Users

All participants positively evaluated the screen designed to simulate the location of other app users (Figure 3D). One main benefit of this screen was to support an awareness that other female forces veterans were also using the app. This, in turn, led to motivating participants and supporting continued engagement.

*I didn’t realize there was that many people using it around my area. If there’s that many people using it, it motivates me to maybe use it more because others are clearly using it as well*.

It was also considered that this screen would enhance engagement by creating a sense of community between female forces veterans. This sense of community helped participants recognize that their difficulties were shared by others, which may have reduced feelings of isolation and encouraged help-seeking behavior.

*Adds a sense of community. Encourages you to believe you’re not the only one* [female forces veteran] *facing mental health difficulties. In this sense it motivates you*.

The potential benefit of developing a map to enable users to contact each other highlights the importance of peer support in promoting mental health and the need for having this conversation.

*Would it be possible to contact people who have started using the app? I would be really interested in knowing if I could meet them*.

#### Support Preferences

All focus group members highlighted the importance of having some form of human support available. They suggested that support could promote and maintain engagement with the app and be beneficial, especially where users are struggling.

*Some human help might be useful, especially if after two weeks you’ve stopped engaging with the app as you are having problems*.

While providing face-to-face support was highlighted as a potential opportunity to further enhance and maintain engagement, it was also considered that some participants may need it to be offered.

*Face to face is a level of support that people ask for, but they don't actually know how to ask, so being offered it would make it easier, especially when struggling*.

Telephone support also emerged as an acceptable support modality. However, some considerations were raised regarding ways to provide and access it.

*Regarding telephone calls, many don’t answer calls from strangers, so if this were to be offered it might work better if a text was sent prior to a telephone call to advise that it* [the call] *is imminent*.

Furthermore, providing peer support from other female forces veterans was raised as having considerable potential with good levels of acceptability.

*...peer to peer support is very underused but has a positive impact for us female veterans if the right person with similar experiences to us was in the role*.

### Phase 5 (Acceptability)

Median (IQR) values across each of the MAUQ subscales were as follows: ease of use 1.80 (1.20‐2.80), interface and satisfaction 2.50 (1.54‐3.33), and usefulness 2.75 (1.75‐4.00). Note that the lower values indicate higher agreement. Each subscale was further examined to determine percentage agreement with each statement (Table 3). Responses to some items were missing, resulting in varied sample sizes across items (n=19‐20). Overall, most participants agreed with each statement, indicating that the app was highly acceptable, except for “I could use the app even when the Internet connection was poor or not available,” rated by 63% of participants as neutral or disagreed.

**Table 3.** Agreement with each statement.

	Strongly agree/agree	Neutral	Strongly disagree/disagree
Ease of use, n (%)			
The app was easy to use.	17 (85)	0 (0)	3 (15)
It was easy for me to learn and use the app.	19 (95)	1 (5)	0 (0)
The navigation was consistent when moving between screens.	19 (95)	0 (0)	1 (5)
The app interface allowed me to use all the functions offered by the app (such as entering information, responding to reminders, and viewing information).	18 (90)	0 (0)	2 (10)
Whenever I made a mistake using the app, I could recover easily and quickly.	12 (60)	7 (35)	1 (5)
Interface and satisfaction, n (%)			
I like the interface of the app.	17 (85)	2 (10)	1 (5)
The information in the app was well organized, so I could easily find the information I needed.	14 (70)	2 (10)	4 (20)
The app acknowledged and provided information to let me know the progress of my action.	18 (90)	1 (5)	1 (5)
I feel comfortable using this app in social settings.	14 (74)	3 (16)	2 (10)
The amount of time involved in using this app has been fitting (adequate) for me.	13 (69)	1 (5)	5 (26)
Overall, I am satisfied with this app.	12 (60)	1 (5)	7 (35)
Usefulness, n (%)			
The app would be useful for my health and well-being.	11 (58)	2 (10)	6 (32)
This app has all the functions and capabilities I expected it to have.	14 (70)	4 (20)	2 (10)
I could use the app even when the Internet connection was poor or not available.	7 (37)	9 (47)	3 (16)
The app provides an acceptable way to receive mental well-being services and allows me to track my own activities.	15 (75)	2 (10)	3 (15)

Using a 7-point Likert scale (1=Strong Agree to 7=Strongly Disagree) a descriptive analysis was undertaken on questions related to 5 main areas associated with IonaFFV. Participants considered the language engaging (mean 1.85, SD 0.87), SOS button was required in the event of a mental health crisis (mean 1.95, SD 1.47), and the signposting information included within IonaFFV was helpful (mean 2.50, SD 1.38). However, it was considered that signposting details to regional organizations should be provided (mean 2.10, SD 1.15). Participants also indicated a need for human support to engage with the app (mean 1.90, SD 1.18). In follow-up questions about human support, where participants could select more than 1 option, there was a preference for weekly scheduled support provided by a professional or voluntary or community sector staff over the telephone (Table 4).

**Table 4.** Support preferences (N=19).

Questions and responses		Frequency, n (%)
Type of human support		
	Weekly contact with a professional	10 (53)
	Weekly contact with voluntary or community organization staff	10 (53)
	Weekly contact with a peer	9 (47)
	On-demand contact with a peer	8 (42)
	On-demand contact with a professional	5 (26)
	On-demand contact with voluntary or community organization staff	4 (21)
Ways human support should be offered		
	Telephone	14 (74)
	Videoconference	13 (68)
	Text	11 (58)
	Face-to-face	8 (42)
	Email	8 (42)
Types of organization(s) to offer human support		
	NHS (veteran)	16 (84)
	NHS (nonveteran)	15 (79)
	Voluntary and community sector (veteran)	12 (63)
	Voluntary and community sector (nonveteran)	9 (47)
	Workplace assistance program	8 (42)
Areas for human support to cover^a^		
	Motivation	14 (78)
	Process accountability (support engagement with CBT^b^ techniques)	14 (78)
	Goal setting	12 (67)
	Signposting opportunities	10 (56)
	Expectations	9 (50)
	Performance monitoring	8 (44)

a N=18.

b CBT: cognitive behavioral therapy.

Interestingly, while there was least preference for weekly support to be provided by peers, using peers was identified as the most preferred in the event support was provided on demand. Where support was provided, it was considered that it should be focused on motivating continued engagement and supporting engagement with techniques used within the app as required.

## Discussion

### Principal Findings

Overall, adopting CBT as the basis of an app to support low mood and worry management in female forces veterans was acceptable. Participants found that the CBT model adapted to reflect female forces veterans both aided understanding of their emotional difficulties and helped tailor the app to them as a distinct group. Furthermore, there was good engagement with techniques used to support low mood and worry management, with initial engagement further enhanced by highlighting use of IonaFFV by other users at the start of engagement and the contextual factors. However, participants differ from most previous research studies with respect to the following characteristics: from the Navy, underwent unplanned discharge, and deployed. Although the average age is consistent with previous research examining access to the Op COURAGE high-intensity service [10].

Applying the EVF [43] to inform the development of an artificial intelligence–supported CBT app for the management of low mood and worry was, however, important to enhance acceptability and usefulness among female forces veterans. Analysis of phases 2 and 4 highlighted the need to make adaptations to ensure that IonaFFV specifically represented their transition from serving into civilian life. Focusing on the EVF context dimension highlighted the need to redirect adaptations from serving to female forces veterans to adequately capture this transition while maximizing acceptability. This was exemplified by reactions to the term “ex-servicewomen,” which was not considered to represent a new civilian identity while acknowledging their past service in the armed forces. This highlights the importance of the language dimension of the EVF when considering adaptations for female forces veterans.

Of interest, “ex-servicewomen” was the term originally proposed during phase 1 of the development process comprising senior members of the armed forces community and service providers. Furthermore, while phase 1 was used to identify the graphics and voices for use by the avatars, these were not considered to represent female forces veterans as a group and therefore removed during development. Adaptations were also required to enhance usability with respect to the structure of the SOS page, enhance navigation through the app where there was a desire to change CBT technique, and enhance the way in which progress was captured.

### Comparison With Previous Research

Consistent with previous research, the findings of this study reinforce the significant role that serving in the armed forces plays in shaping the identities of both male and female veterans [56]. The results emphasize the particular challenges faced by female forces veterans in negotiating their identities during the transition to civilian life [6]. This highlights the need for tailored approaches that acknowledge their unique experiences in the armed forces. While female forces veterans are often “hypervisible” during their military careers, they may simultaneously suppress behaviors traditionally considered feminine [7]. Failure to recognize these adaptive strategies supports the need identified in this study for appropriate modifications to IonaFFV. Implementing adaptations was recognized to improve acceptability by better reflecting the experiences of female forces veterans as they transition from life in the armed forces to becoming a civilian [6].

While the initial adaptations to IonaXSW used graphics aligned with the armed forces to enhance engagement, participants emphasized the importance of gradually reducing the focus on the armed forces as they progressed through the app to better reflect civilian life [7]. They also recommended replacing quotes associated with the armed forces context with those drawn from civilian life. A lack of recognition of the transition from military to civilian identity could have hindered the process of “rediscovery” that supports successful reintegration [7]. Furthermore, failing to change the term “ex-servicewomen” [1,7], proposed in adaptation phase 1 to female forces veterans, would have further reflected a transition oversight. However, the preference to maintain “veteran” is not consistent with previous research [57], where it is traditionally considered to reflect previous generations of male veterans [57]. Potentially, these differences have arisen from study demand characteristics, given that recruitment materials adopted the term “veteran” [58].

Features of IonaXSW that did not require adaptation are consistent with those commonly identified as acceptable with CBT [59]. For example, the CBT model both enhanced understanding regarding the nature of the mental health difficulty faced and aided an understanding of the way the low-intensity CBT technique may help [60]. Furthermore, consistent with a metasynthesis of computerized CBT, personalization of CBT-specific factor content enhanced relevance [61], promoting connection and engagement. The screen simulating the location of other female forces veterans also served to normalize mental health support [62] and encouraged lateral social comparisons [63].

While the chatbot successfully maintained user engagement with IonaXSW, consistent with findings from CBT-based self-help interventions more broadly, participants continued to express a preference for human support [64]. Among the various forms of support considered acceptable, the most preferred option was scheduled weekly sessions with a professional from a veteran-specific NHS service. This finding suggests potential benefits of integrating support for IonaFFV through services such as Op COURAGE [10]. However, despite the overall acceptability of this service [10], its evaluation included only male veterans, which limits the generalizability of the findings to female armed forces veterans.

Alternatively, nearly half of the participants preferred on-demand peer support, indicating potential advantages similar to those observed with peer-support groups [65]. Implementing IonaFFV alongside support from female forces veterans within veteran-specific services [9] could serve to enhance help-seeking and address existing barriers to engagement [66]. Moreover, as low-intensity CBT techniques can improve access to care, adoption could enable services to manage increased demand without extending waiting times [67]. While IonaFFV incorporates in-app support to promote engagement [68], incorporating peer support could mitigate low levels of engagement with a stand-alone app following download [69].

### Strengths and Limitations

Adopting an approach toward adaptation comprising focus groups to guide initial modifications and using the MAUQ [53] to assess acceptability offers a means to evaluate how well these adaptations are acceptable to a broader group of female forces veterans. This methodology could help address challenges related to the limited generalizability of focus group data [70]. However, the small number of participants completing the MAUQ limited further analysis regarding the acceptability of IonaFFV across different demographic groups. Specifically, only a single participant had served in the Royal Air Force, identified as non-White, or had experience as a reservist. Recruitment challenges for phase 5 may have been influenced by the requirement for participants to engage with IonaFFV for 4 weeks before completing the questionnaire. Therefore, future research should consider strategies to enhance recruitment and questionnaire completion before assessing the acceptability and usability of IonaFFV and generalizing study results [71].

It may also have been of benefit to collect additional information in phase 5 regarding the service experiences of female forces veterans. Female forces veterans experience higher levels of in-service adversity than their male colleagues, including exposure to military sexual trauma [72]. Although IonaFFV was explicitly designed for female forces veterans experiencing low mood or difficulties with worry management, some questionnaire responses may have been influenced by past service-related adversity. This possibility was suggested by a free-text questionnaire response which indicated that the participant may have been experiencing moderate to severe mental health difficulties. Gathering more comprehensive data on participants’ service experiences could have supported a deeper interpretation of the findings and informed further adaptations, such as incorporating broader signposting information.

### Conclusions

There is a clear need to recognize female forces veterans as a distinct group from their male peers. While sharing many values and traditions common across the armed forces, they also hold a collective identity that differs from that of male veterans [73]. Addressing the current inequities in access to, and appropriateness of, mental health services for female veterans [8,14] requires a focus on enhancing the acceptability of veteran-specific services. In particular, using dimensions of the EVF to structure the focus group topic guide was valuable to identify the types of adaptations necessary to maximize acceptability. The EVF also helped highlight how these dimensions shift during the transition from serving to armed forces veteran, offering insight into how mental health services can be better tailored for female forces veterans. When applied to the development of a CBT-based mobile phone app for managing low mood and worry, the EVF informed adaptations that produced a digital mental health approach with strong usability, acceptability, and perceived usefulness among female forces veterans.

## Abbreviations

CBT: cognitive behavioral therapy
EVF: Ecological Validity Framework
IonaFFV: Iona Female Forces Veterans
IonaXSW: Iona Ex-Servicewomen
MAUQ: mHealth App Usability Questionnaire
NHS: National Health Service
